# A Plea for the General Use of Measures to Prevent Ophthalmia Neonatorum

**Published:** 1897-01

**Authors:** Alvin A. Hubbell

**Affiliations:** Buffalo, N. Y.,; Professor of ophthalmology and otology, Medical Department of Niagara University, 212 Franklin Street


### A PLEA FOR THE GENERAL USE OF MEASURES TO PREVENT OPHTHALMIA NEONATORUM.

By ALVIN A. HUBBELL, M. D., Ph. D., Buffalo, N. Y.,
Professor of ophthalmology and otology, Medical Department of Niagara University.

THE importance of a remedy is measured by the seriousness of the disease which it prevents or cures ; and a preventive remedy is more valuable than a curative one in proportion to the amount of injury and fatality saved. If vaccination prevents individuals from having small-pox, or from having it in a severe or fatal form, while with all known remedies applied to the cure of the unmodified disease a large proportion die, the importance of the prevention immeasurably outweighs that of the cure. If, by the adoption of efficient sanitary measures, Buffalo's death-rate is reduced one-half, and that city becomes perhaps the healthiest of its size in the world, the importance of the measures which prevent disease ranks high above the best remedies that can be used to cure it.

If the sad results of purulent conjunctivitis of infants can be averted manyfold by preventive treatment, in so much is the importance, here, of prophylaxis magnified over curative treatment.

I have set myself the task of showing that such is the case, and I invite the profession to reconsider the attitude which should be taken toward this subject.

In the first place, purulent conjunctivitis of infants, or ophthalmia neonatorum, is a most serious disease of the eyes. Statistics show that of those who have the disease and have not been properly treated, from 10 to 15 per cent. go blind in both eyes ; of those in whom the treatment has been in part what it should be, from 4 to 8 per cent.; and of those with the most skilful treatment, from 1 to 4 per cent. As a large proportion having the disease are, without doubt, indifferently treated, it is probable that in every 100 such cases at least five lose their vision in both eyes. A much larger proportion become blind in one eye.

In the second place, it is a frequent disease. Both old and recent statistics give ample proof of this. From them we learn that in institutions for the blind there are, according to Magnus, in Europe, 24 per cent. blind from this disease ; in Switzerland, 26 per cent. ; in Germany, 26 per cent. ; in Hungary, 21 per cent. Reinhard estimates this class of inmates in the asylums of Ger-

1. Read before the New York State Medical Association, October 14, 1896.

many, Austria and Holland at 40 per cent., while Horner places it for Germany and Austria at 33 per cent., both estimates being much larger than those of Magnus.

In the asylums of cities the following percentages have been found: Copenhagen (Haussman), 8; Vienna (Haussman), 20; Berlin (Haussman), 20; Berlin, (Katz, at another time,) 41; Paris (in 208 young persons blind), 45; Paris (Claisse), 46; Breslau (Magnus), 34; Sheffield (Snell), 38; New York, Batavia, Philadelphia, Chicago, (Andrews, Howe, Harlan, Prince,) about 20. In the eye hospitals and dispensaries among those blind from this cause there were in nine German hospitals (Magnus), 10.8; Breslau (Cohen), 11.1; Paris (Dumas), 69.3; Paris (Bourjot St. Hilaire), 27.

Among the whole blind population of Brunswick, Fuchs has estimated that 28 per cent. were blind from this disease, and of that of Nassau, 13 per cent. Bell (*British Medical Journal*, March 3, 1888,) claimed that there were 72 per cent. among those of England. The census of the United States (May) gives a proportion of 20 per cent.

Approaching this question as to frequency from another point of view, we find important testimony when considered in its relation to the whole number of births in a city or country. After a careful inquiry, Widmark (*Rev. d' Ophtalmologie;* and *Sajous' Annual*, 1889, Section B, page 167,) concluded that three out of every 1,000 children born in Sweden had ophthalmia neonatorum. Skrebitzky (*St. Petersburg Med. Woch.*, June 8, 1887,) estimated that among the poor classes of St. Petersburg 8 to 12 per cent. of the new-born were afflicted with this disease. Calculating that for every one blind from this disease about fifteen to twenty others have had it and been subjected to the frightful risks of blindness, but who have fortunately recovered, we may reasonably conclude that in the United States from one to two out of every 1,000 children have been victims of this disease. My own inquiry among physicians of Buffalo leads me to believe that in every 1,000 infants born in that city at least five have this disease. In the practice of some physicians of my acquaintance the proportion is greater than this, while in others it is less. In European countries the statistics show that the proportion is greater than in the United States.

We have another index of the frequency of ophthalmia in the reports of the various clinics. Mules (Manchester, England,)

reports a total of 97,237 eye-cases attending the Manchester Eye Hospital from 1876 to 1884, inclusive, of which 3,319, or over 3 per cent., were ophthalmia neonatorum. Hirschberg reports, in 21,440 cases, 314, or nearly 1.5 per cent.; Schoeler, in 10,000 cases, 156, or over 1.5 per cent.; Horner, in 10,000 cases, 161, or 1.6 per cent. In the eye hospitals of the United States the proportion varies, probably, from 0.4 to 1 per cent.

The maternity hospitals furnish additional information, although it must be admitted that the frequency is much greater here than in private practice. But, without special prophylaxis, it has been found that from 1 to 5 per cent., and sometimes as high as 10 per cent., of the infants born in these institutions have ophthalmia.

These studies of statistics force us to conclude that ophthalmia neonatorum is not only a most severe disease and dangerous to sight, but that it is a common affection. Its consequences are so grave, both to the individual and public economics, that its proper treatment has been emphasised in such a manner that it becomes by law a misdemeanor, punishable by severe penalty, in this and other states, for the nurse or midwife not to report the earliest symptoms to a physician. This great stress is laid upon its cure, and yet, under the most skilful treatment, even early begun, many eyes go blind. The lessening of its frequency is none the less desirable, and the importance of preventive measures is none the less apparent, but such measures have not until recently been developed and made efficient and available. The time has, however, now arrived when the greater emphasis should be placed where it always belongs—namely, on the prevention, rather than on the cure.

The prevention of ophthalmia neonatorum is not altogether a recent study. Recognising the fact that the disease is caused by infection from the secretions of the parturient canal, vaginal douches previous to and during labor have been recommended and practised by various physicians for many years. This practice is commendable, is measurably effectual, but it has never become common. With the development of the germ theory of disease and the discovery of germicides came a new interest in this affection. The gonococcus of Neiser, of Breslau, described by him in 1879, (*Centralblatt für Med. Wiss.*, No. 28,) became an objective entity and cause in gonorrhea, and gonorrhea was thought to be the prime source of ophthalmia neonatorum. Here, then, was something to be either eliminated or killed, and with one or both of these objects in view a scientific prophylaxis has been attempted and put into practice. Vaginal douches and cleansing the eyes were again resorted to with increased zeal, water, both with and without antiseptics, being used. But it was not till 1883 that Credé, of Leipsic, after experimenting with different applications and substances and solutions of different strengths, brought to the attention of the profession a specifically germicidal method of prophylaxis. He supported his claims for the method by an experience which has since been verified by all who have practised it with the same care. So potent has been this practice that where-ever it has been used properly, the occurrence of ophthalmia neonatorum has been reduced from the percentages prevailing in institutions, such as I have already named, to 1 or 2 per cent., and in some institutions almost to zero. I refer to institutions, because it is here that the merits of the method can best be tested and comparisons best made.

Credé's method is as follows: The child is put into the bath immediately after ligating the umbilic cord, the eyes are carefully cleansed with a linen cloth, or better still, absorbent cotton, and then the eyelids are slightly separated and a single drop of a 2 per cent. (10-gr.) solution of nitrate of silver is instilled between them. In order to make secure the instillation of a *single drop* and to avoid the irritation which follows the application of a larger quantity, Credé recommended the use of a glass rod 15 centimeters (6 inches) long and 3 millimeters ($\frac{1}{8}$ inch) in diameter, with smooth, rounded ends, from which to let fall upon the cornea a single drop of the solution (Carl F. S. Credé, *Die Verhuntung der Augentzundung der Neugeborenen*, *Archiv. für Gynakologie*, Vol. XXI., 1883). He condemned drop-bottles as a means of applying the silver and did not evert the lids. He at first washed the eyes before the instillation with a 2 to 200 solution of salicylic acid and afterward applied compresses over the eyes wet with the same. But this was soon discarded, as well as the vaginal douches during labor. He at last used nothing but plain water with which to wash the eyes before the instillation. The efficiency of Credé's method is more explicitly shown by the following statistics:

In Credé's own institution the percentage in 1880 to 1883 (including 1,160 births with only 1 or 2 cases) fell from 10.8 to 0.1 to 0.2. In 1886 he reported 1,211 births with 3 slightly affected, or 0.25 per cent.

In the Greifswald gynecological clinic the cases of the disease were reduced from a per cent. varying from 9 to 22.4 to 1.8 ; in the Berlin Charité, from 9 to 0.9 ; in Königstein's, to 0.7 ; in Krunkenberg's, to 0.14 ; in Felsenreich's, from 4.3 to 1.5 ; in A. Russell Simpson's, from 11.76 to 5 ; in Bayer's, from 12.3 to 0 (Fuch's, On blindness).

Giales (*New Orleans Medical and Surgical Journal*, 1886,) stated that in the foundling hospital of Paris some twenty years ago the percentage sometimes reached as high as 80 or 90 ; but today, under Credé's method, it was not far from zero.

Levan (*Sajous' Annual*, 1889, B, page 66,) used vaginal injections of 1 to 2,000 bichloride of mercury as a prophylaxis in 30 cases, and 5 of the infants became affected with mild ophthalmia. In 100 cases no injections were used, but Credé's method was followed, and not a single case of the disease occurred. In one other case in which the silver solution instillation was inadvertently omitted in one eye, that eye became affected, while the one receiving the instillation escaped.

Steinbüchel (*Wiener Klin. Woch.*, Vienna, 1891,) says that during a period of two years preceding 1891, 5,994 children were born in Vienna hospitals, and the eyes became affected in only 1.75 per cent., while before the Credé method it was much higher.

Dr. J. Lwow-Kasan (*Deutsche Med. Zeit.*, 1889,) reported in 1889 914 infants treated by Credé's method without a single case of the disease.

Dr. May (*Medical Record*, 1895,) says that in 4,000 births at the Sloane Maternity in New York during six years, by Credé's method, no ophthalmia had occurred. At the Nursery and Child's hospital, New York, there had been 563 births during 1891 to 1894 with but one case. At the New York Maternity hospital, Blackwell's Island, the disease was "very uncommon" among 300 births annually.

Dr. Garrigues (*American Journal of Medical Sciences*, 1884, Vol. LXXXVIII., page 444,) during his service in the New York Maternity hospital in 1882 to 1884, followed Credé's practice in 351 infants and not one was affected with ophthalmia. One other case was delivered in the absence of the house surgeon and the silver was neglected, and this child had the disease and lost both eyes in spite of special treatment. In Dr. Mundé's service in 1885 in the same institution, 83 infants received Credé's treatment and not one had ophthalmia. At a previous period, according to the house surgeon, Dr. Pierson, "it had been no uncommon occurrence to have half a dozen of these cases on hand in a service of 35 confinements a month."

Without deducing further experience this seems sufficient to justify the method of Credé as a preventive to ophthalmia neonatorum. But other methods have been brought forward, and although not so extensively corroborated, make claims which merit consideration. For example, Dr. Pinard, of Paris, in 1888 introduced the practice of squeezing the juice from a citron and dropping it directly into the eyes of every child as soon as born, with encouraging results. At the Baudelocque clinic in 1891, out of 1,615 births there were 13 cases affected, or less than 1 per cent. (*Panas Maladies des Yeux.*, T. II., page 215.) In 1890 he had used these instillations in 852 infants, of whom 14, or 1.64 per cent., had ophthalmia. Recently Pinard has substituted for the citron juice a solution, 5 to 100, of citric acid.

Dr. E. Valude, of Paris, in 1891, brought forward iodoform insufflations into the eyes of the new-born as a prophylactic. (*Annales d'Oculistique*, T. 106, 1891, page 96.) His method is as follows: Immediately after birth and before tying the cord, the lids are gently dried with cotton impregnated or not with an antiseptic solution, and all greasy matter is removed from the lashes and edges of the lids, and then the lids are slightly separated and a certain quantity of finely powdered iodoform insufflated or dusted into the eyes. It is not necessary to repeat the insufflation. He first began this practice in the Hospital Saint-Louis, in 1890, in the service of Dr. Bar. From December 1, 1890, to April 1, 1891, 264 infants were thus disinfected, of which 13, or nearly 5 per cent., had ophthalmia. Credé's method had been previously used in the same place and under the same conditions with a result of 7 to 8 per cent. In Dr. Tarnier's service the use of the iodoform insufflations gave the same comparative results. In 248 infants there were 5 cases of the disease, or 2 per cent., while previously in 218 treated by Credé's method 13, or nearly 6 per cent., became affected.

Madame Henry, midwife-in-chief of the Maternity of Paris, washed the infant's eyes at birth, and twice a day for a time afterward, with 1 to 1,000 solution of bichloride of mercury, and in a long series of deliveries did not get a single case of the disease. (Valude, *les Ophtalmies du Nouveau-ne*, Paris, 1895, page 29.)

With carbolised water, Königstein, Olshausen and others reduced the frequency of the disease to 1 to 4 per cent. Others have had success with chlorine water, with solutions of formalin, of permanganate of potash, of beta naphthol, of boracic acid, of bichloride of mercury and of other substances. Ordinary water alone has its advocates. Ludwig Korn, in the obstetrical clinic of Dresden, reports much success by conscientiously cleansing the infant's lids and their surroundings with water before opening the eyes. Simeon Snell (*London Lancet*, April 25, 1891,) has adopted the same method in the Jessop Hospital, at Sheffield, England, and in 2,000 infants treated in this way there has not been a case of ophthalmia.

Such experience as this, and much more of a similar character which could be cited, puts beyond question the efficiency of the prophylactic treatment of ophthalmia neonatorum. Then, if this is a frequent disease, if its results even under treatment are deplorable, and if preventive measures diminish its frequency from eight to ten fold, thus diminishing in the same proportion the suffering, misery and loss of money both to the individual and state, by just so much is the importance of prophylaxis in this disease to be weighed and measured. If the disease could be diminished but one-half, its value could not be overestimated, and its practice could not be too stringently enforced. But while it is true that we have these invaluable measures, yet the profession at large is, I fear, indifferent or uninformed regarding them. The physician is not unmindful of the burdens and deprivations of blindness; then he ought to be alive to the need of averting it, and on the alert to use every means toward removing the causes that lead to it. Next to life, sight is the most precious thing on earth. Granting, then, that prophylaxis demands a full and general recognition, which method is preferable?

There is no doubt that contagion can be washed away either from the vagina of the mother or the eyes of the infant, if scrupulously and diligently done, and that even ordinary water will suffice. Were this process of cleansing the mother's vagina and the child's eyes so simple that it could be used under any circumstances, it might be chosen; but it is not. Vaginal injections require intelligent supervision and at least a few special appliances and conveniences in order to be well done, and they are not always at hand. A certain amount of trouble also attaches to their use and their necessary repetition, and even those competent to administer them are disposed to be indifferent and omit them entirely, or, at best, resort to them slightingly. The thorough and proper washing of the infant's eyes is also attended with an amount of concern that requires an intelligence not always present. Hence, vaginal injections or washing the infant's eyes have drawbacks that render them inapplicable as a general and trustworthy prophylactic.

The contagium producing ophthalmia can also be destroyed by certain substances, even without preliminary vaginal douches or searching washings of the eyes, and their application is easy and simple. Those substances which require large dilution, like carbolic acid, boric acid, salicylic acid, permanganate of potash, bichloride of mercury, formalin and others, lose much of their power in such dilution as the eyes will bear, and must be looked upon in this form more as washes than germicides, and they, too, require to be properly prepared and carefully and intelligently administered to be effectual. They have, then, requirements that are liable not to be met, and they cannot be uniformly serviceable in all hands. Hence, germicides in other forms must be sought whose application is more simple and easy. It has been shown that iodoform justifies, at least measurably, the claims that have been made for it, and the method of using it which is followed by Valude, and which has already been described, can be practised by anybody. Should the results so far published be verified in the future, it has this to commend it, that, by its odor and appearance it cannot readily be mistaken for another drug, it is easily obtained and its use causes no marked irritation of the infant's eyes or injury to those handling it. The most objectionable feature it possesses is its offensive odor, and this is of no consequence in comparison with the benefits expected from its use.

Dr. Weeks and others have demonstrated that nitrate of silver is one of the most powerful germicides known. Unfortunately its staining qualities render it inapplicable for general surgical use. But, within a limited range, it has a usefulness that cannot be equaled by any other known substance. As a germicide in the eye its power has been repeatedly demonstrated, and not the least in ophthalmia neonatorum, both as a preventive and curative remedy. The experience that I have already cited indubitably proves that by Credé's method it has such power, and that no special or painstaking washings preliminary to its use are strictly necessary. Having such power, then, and having such simplicity of application to commend it, together with the marvelous results which have been obtained in support of it, why should not this substance and this method become universal? It is harmless to the infant's eyes, the irritation which it excites being but mild and temporary, and the inexperienced, as well as the nurse, the midwife or the physician, can do so simple a thing as is required in its application.

As to when prophylactic measures should be used there may be difference of opinion. All will agree, however, that they should be used in cases where the mother is known or suspected to have gonorrhea. But the disease often occurs where there is not the slightest history of maternal gonorrhea, where by reason of symptoms or position in society there is no ground for suspicion of its presence. Indeed, there is abundant evidence that cases now and then arise without gonorrhea. Neither is the disease limited, by any means, to those in the lower walks of life, as every practitioner of experience knows.

How, then, and by whom are the suspicious cases to be distinguished from the unsuspicious, the infective from the noninfective? Even if it were true that the cause is always gonorrhea, only a bacteriological examination of the vaginal secretions for the gonococcus would determine its presence and distinguish a gonorrheal from a noncontagious vaginitis ; hence such examination would generally be impracticable, and, if practicable, would be useless because of the loss of time required in making it. The history of a case and the presence of symptoms of gonorrhea certainly render it suspicious, but these are often suppressed or masked or cannot be elicited, even by shrewd and inquiring attendants.

It being impracticable, then, to at once and always recognise or determine the presence of the causative principle of the disease, it being true that even the most wide-awake and skilful practitioner, and surely the average midwife, cannot tell when or in what station of life the disease may occur, it seems to be conclusive that there is but one course to pursue, and that is to apply some prophylactic treatment to the eyes of every living infant as soon as practicable after birth. The sequences of ophthalmia neonatorum are too serious, its occurrence is too frequent, and the prophylaxis, particularly Credè's method, so certain, that it is absolutely wrong to neglect it.

Furthermore, if legal obligations placed upon the early recognition and treatment of the disease are found to be necessary, how much more important is it that legislation should make compulsory and general a prophylaxis which insures to public and private good many times the benefits derived from the most skilful and potent treatment of the disease known after it has developed.

212 Franklin Street.

